# Cone-Beam Computed Tomographic Evaluation of Variant Foramina of the Clivus: A Retrospective Study From a Tertiary Dental Institution in South India

**DOI:** 10.7759/cureus.111482

**Published:** 2026-06-25

**Authors:** Sanjana MR, Chandrasekaran Krithika, KV Rajasekhar, Ragu Ganesh M

**Affiliations:** 1 Oral Medicine and Radiology, Meenakshi Academy of Higher Education and Research (MAHER), Chennai, IND; 2 Radiology, Meenakshi Medical College Hospital and Research Institute, Kanchipuram, IND; 3 Oral Medicine and Radiology, Ragu Dental Clinic, Chennai, IND

**Keywords:** canalis basilaris medianus, clivus, cone-beam computed tomography, craniopharyngeal canal, fossa navicularis magna

## Abstract

Introduction: Anatomical variants involving the clivus, including Canalis Basilaris Medianus (CBM), Craniopharyngeal Canal (CPC), and Fossa Navicularis Magna (FNM), are uncommon developmental osseous defects that are usually identified incidentally during radiographic examination. Although generally asymptomatic, these variants may mimic pathological lesions or fractures and may possess important clinical and surgical implications. Large field-of-view cone-beam computed tomography (CBCT) scans provide an opportunity for incidental identification of such variants during routine dental imaging. The present study aimed to evaluate the prevalence and demographic distribution of anatomical variant foramina involving the clivus in archived CBCT scans obtained from a tertiary dental institution in South India.

Materials and methods: This retrospective observational study was conducted using 50 archived large field-of-view CBCT scans retrieved from the Department of Oral Medicine and Radiology, Meenakshi Ammal Dental College and Hospital, Chennai. CBCT scans were evaluated in axial, coronal, and sagittal sections for the presence of CBM, CPC, and FNM. Demographic distribution and prevalence of variants were assessed using descriptive statistics, and associations between variables were evaluated using the Chi-square test and Fisher’s exact test.

Results: Clival anatomical variants were identified in 20 (40.0%) subjects. CBM was the most frequently observed variant (12, 24.0%), followed by CPC (5, 10.0%) and FNM (5, 10.0%). CBM demonstrated a relatively uniform age distribution, whereas CPC showed a statistically significant association with age group (p = 0.033). FNM was identified only among male subjects in the present sample, although this association did not reach statistical significance. Co-occurrence of two variants was identified in two (10.0%) positive cases.

Conclusion: Clival anatomical variants may be encountered as incidental findings on large field-of-view CBCT scans and can be identified during routine radiographic evaluation. Adequate knowledge regarding their radiographic appearance is essential to avoid misdiagnosis and facilitate accurate CBCT interpretation, appropriate referral, and effective preoperative planning. Given the relatively small sample size and single-center design, further studies are required to better characterize the prevalence and clinical significance of these variants.

## Introduction

The sphenoid bone is a centrally located osseous structure forming a major component of the skull base [1]. It is bounded anteriorly by the frontal and ethmoid bones, laterally by the temporal bones, and posteriorly by the occipital bone. Anatomically, the sphenoid bone comprises four major parts: the body, greater wings, lesser wings, and pterygoid processes [1]. The body of the sphenoid contains the sella turcica, a saddle-shaped depression that houses the pituitary gland [1]. Posteriorly, the sphenoid contributes to the formation of the clivus, a sloping bony surface extending from the dorsum sellae to the foramen magnum [2]. The inferior surface of the clivus forms the roof of the nasopharynx and represents an anatomically important region at the skull base [2].

Several anatomical variations involving the clivus and adjacent skull base region have been documented in the literature. Among these, Fossa Navicularis Magna (FNM), Canalis Basilaris Medianus (CBM), and Craniopharyngeal Canal (CPC) are among the most frequently encountered variant osseous canals and foramina. These anatomical variants are generally asymptomatic and are most often identified incidentally during radiographic evaluation [3]. However, due to their radiographic appearance and anatomical location, they may occasionally mimic pathological lesions, fractures, or osteolytic defects, thereby resulting in diagnostic confusion and unnecessary clinical intervention [2,3]. Furthermore, certain variant canals may act as potential communication pathways between intracranial and extracranial structures, thereby increasing the risk of infection spread or hemorrhagic complications during surgical procedures involving the skull base [3].

Evaluation of the clivus is often challenging because of its deep anatomical location and limited accessibility during clinical examination. Consequently, radiographic imaging plays a critical role in the assessment of this region [4]. Conventional imaging modalities such as computed tomography (CT) and magnetic resonance imaging (MRI) are considered reliable methods for evaluating clival anatomy and associated pathologies [4]. Adequate knowledge regarding the normal anatomy and anatomical variations of the clivus is therefore essential for oral radiologists, maxillofacial radiologists, and surgeons to facilitate accurate diagnosis, prevent misinterpretation of anatomical variants, and assist in surgical planning [4,5].

Cone-beam computed tomography (CBCT) has become an indispensable imaging modality in contemporary dental practice because of its high spatial resolution, lower radiation dose compared to conventional CT, and widespread use in craniofacial imaging [5,6]. Large field-of-view CBCT examinations frequently encompass anatomical structures extending beyond the immediate dentomaxillofacial region, including the skull base and clivus [5]. This provides an important opportunity for incidental identification of anatomical variations involving the clivus during routine dental imaging [6]. Recognition of these variants is clinically significant, as it may reduce the risk of misdiagnosis, prevent unnecessary surgical intervention, and provide valuable preoperative information regarding potential anatomical pathways for the spread of infection or surgical complications.

Despite the increasing use of CBCT in dental practice, data regarding the occurrence of clival anatomical variants in Indian populations remain limited, particularly from South India. Improved characterization of these variants may enhance diagnostic accuracy and support appropriate radiological interpretation of incidental skull base findings. Therefore, the present retrospective study aimed to evaluate the prevalence and demographic distribution of anatomical variant foramina involving the clivus in archived large field-of-view CBCT scans obtained from a tertiary care dental institution in South India.

## Materials and methods

The present retrospective observational study was conducted in the Department of Oral Medicine and Radiology, Meenakshi Ammal Dental College and Hospital, Chennai, Tamil Nadu, India. The study was performed using archived cone-beam computed tomography (CBCT) scans retrieved from the departmental database. The archived CBCT scans had originally been acquired for routine educational, diagnostic, and treatment-planning purposes, including orthodontic, implant, and other dentomaxillofacial assessments, and were subsequently reviewed for research purposes. Ethical clearance was obtained from the Institutional Ethics Committee prior to commencement of the study in August 2025 (Reference No.: MADC/IEC/II/93/2025).

The study included CBCT scans acquired over a two-year period from October 2023 to September 2025. Retrieval and evaluation of the archived CBCT scans were carried out between October 2025 and April 2026. CBCT image retrieval and evaluation were performed using the proprietary imaging software available with the CBCT system. Images were assessed using multiplanar reconstruction views (axial, coronal, and sagittal), with adjustment of brightness, contrast, and magnification whenever required to facilitate radiographic interpretation.

The sample size for the present study was determined based on the findings of an unpublished pilot study conducted by the investigators on 30 CBCT scans obtained from a tertiary care dental hospital in Chennai. The sample size was calculated using the formula:

$n = \frac{Z^{2}pq}{d^{2}}$,

where n = minimum required sample size, Z = 1.96 at a 95% confidence level, p = 0.70 (pilot study prevalence), q = 0.30, and d = 0.14 (20% of the estimated prevalence).

Based on these assumptions, the minimum required sample size was estimated to be 43 scans. The prevalence observed in the pilot study was used solely for sample size estimation and was not intended to represent the expected prevalence in the final study population. A total of 50 large field-of-view CBCT scans encompassing the skull base region were included in the study using a consecutive sampling method.

Consecutive archived CBCT scans fulfilling the eligibility criteria during the study period were screened for inclusion. After application of the inclusion and exclusion criteria, 50 large field-of-view CBCT scans were included for final analysis. Where multiple scans of the same individual were available, only one scan was considered to avoid duplication of observations.

The inclusion criteria comprised CBCT scans with complete visualization of the skull base and clival region with adequate image quality for radiographic assessment. Scans belonging to subjects of both sexes and all age groups were included in the study. CBCT scans demonstrating inadequate field of view, poor image quality, motion blur, image distortion, or artifacts obscuring the anatomical region of interest were excluded. In addition, scans showing evidence of osseous pathology, congenital craniofacial anomalies, malignancy, or facial fractures involving the skull base region were also excluded from the study.

All CBCT examinations had been acquired using the standardized imaging protocols routinely employed in the department for large field-of-view CBCT imaging encompassing the skull base region. Only scans demonstrating adequate image quality for diagnostic interpretation were included in the study.

**Figure 1:**
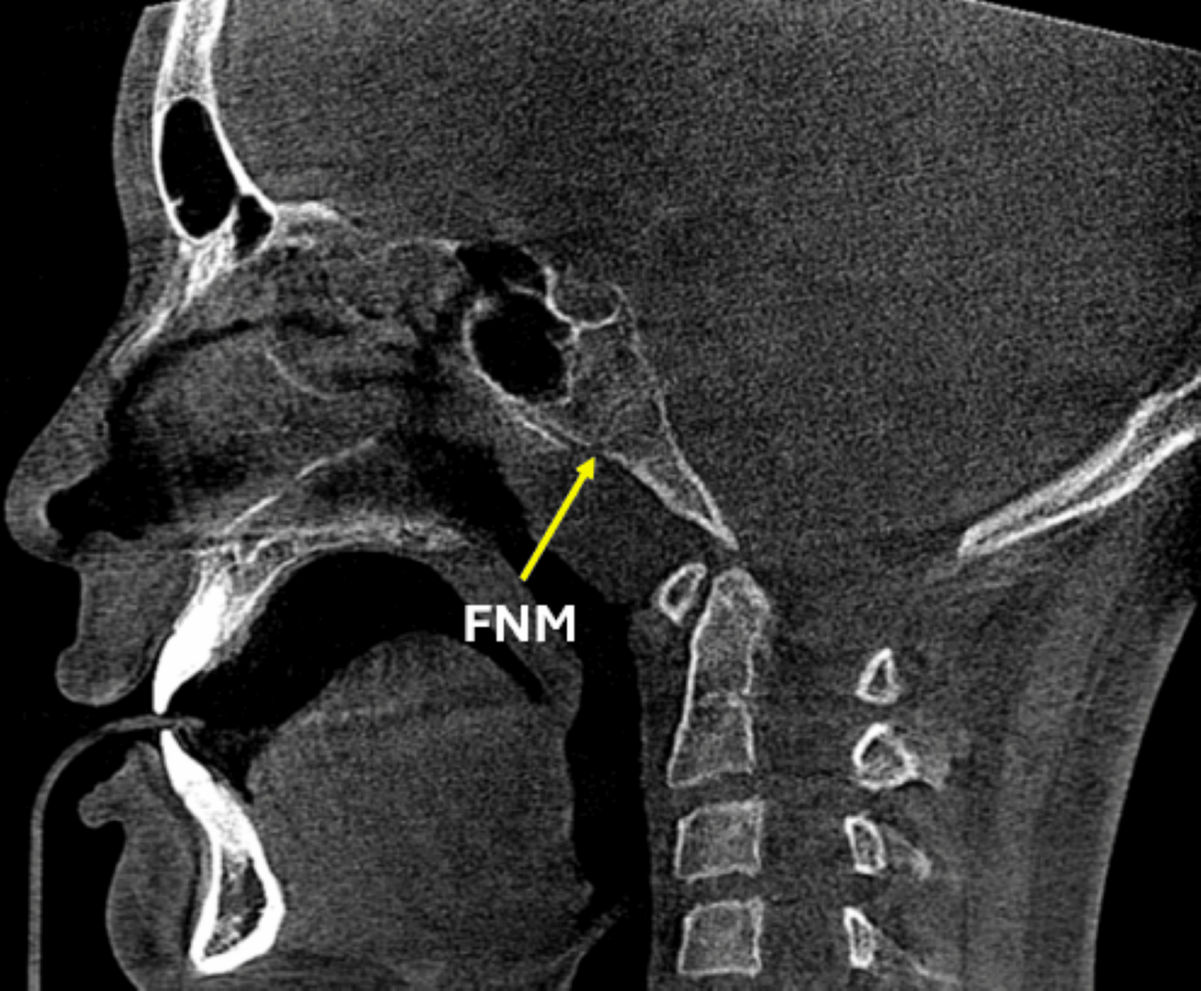
Sagittal CBCT section showing Fossa Navicularis Magna (FNM) as a well-corticated notch-like defect on the inferior aspect of the clivus. CBCT, cone-beam computed tomography.

**Figure 2:**
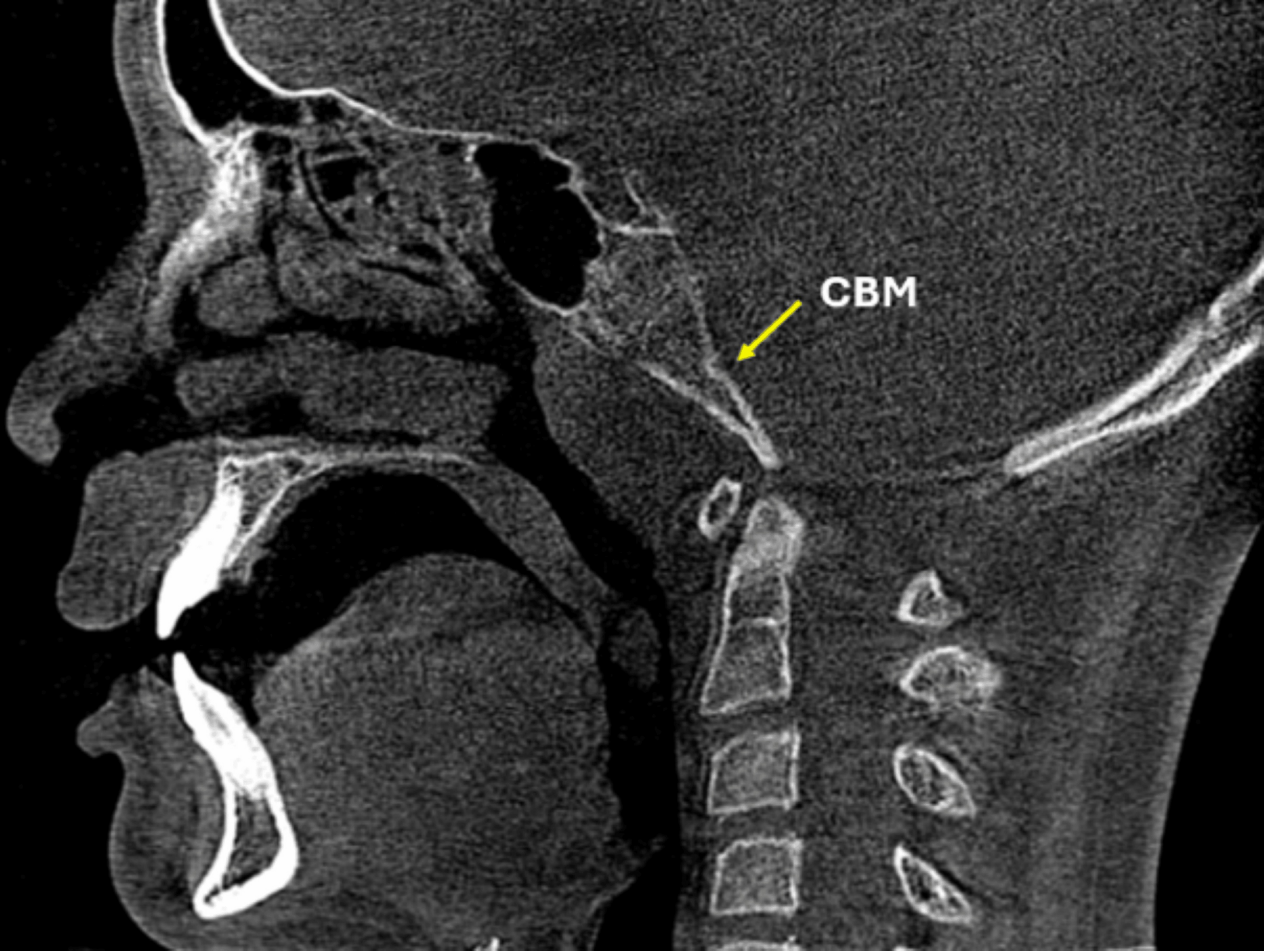
Sagittal CBCT section showing Canalis Basilaris Medianus (CBM) as a radiolucent depression in the basiocciput. CBCT, cone-beam computed tomography.

**Figure 3:**
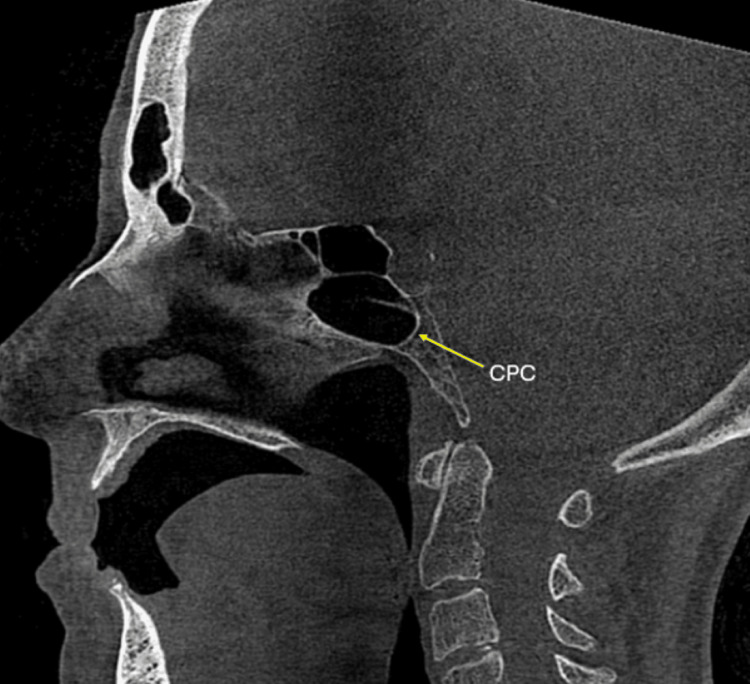
Sagittal CBCT section showing a persistent Craniopharyngeal Canal (CPC) as a corticated tubular channel at the sellar floor. CBCT, cone-beam computed tomography.

All selected CBCT scans were evaluated in axial, coronal, and sagittal sections for the presence of clival anatomical variants. The variants assessed in the present study included Canalis Basilaris Medianus (CBM), Craniopharyngeal Canal (CPC), and Fossa Navicularis Magna (FNM). FNM was identified as a notch-like osseous defect located along the inferior aspect of the clivus and was best appreciated in sagittal sections. CBM was identified as a corticated canal within the basiocciput of the clivus and was predominantly evaluated in axial and sagittal sections. CPC was identified as a corticated osseous tract extending between the sella turcica and the nasopharynx, most clearly visualized in sagittal sections (Figures 1-3).

All CBCT scans were independently evaluated by two experienced oral radiologists. To ensure reproducibility and minimize observer variability, all scans were assessed twice at separate time intervals. In cases of disagreement, the scans were jointly reviewed, and a consensus diagnosis was reached following discussion between the two observers. Interobserver agreement between the two radiologists was assessed using Cohen's kappa statistic. The observations obtained from the CBCT evaluation were systematically recorded in a structured Microsoft Excel (Microsoft Corporation, Redmond, WA, USA) data collection sheet prepared specifically for the study.

Statistical analysis was performed using Statistical Package for the Social Sciences (SPSS) software, version 26.0 (IBM Corp., Armonk, NY, USA). Descriptive statistics were presented as frequencies and percentages. Associations between categorical variables were evaluated using the Chi-square test. Cohen's kappa coefficient was calculated to assess interobserver agreement. A p-value less than 0.05 was considered statistically significant.

## Results

The present study included 50 large field-of-view CBCT scans encompassing the skull base region. The study population consisted of 30 (60.0%) male and 20 (40.0%) female subjects. The age of the subjects ranged from 12 to 75 years, with a mean age of 41.84 ± 17.41 years. Among the various age groups, subjects aged 41-50 years constituted the largest proportion (12, 24.0%), followed by the 21-30 year age group (11, 22.0%). Subjects aged above 60 years accounted for eight (16.0%) cases, while the least represented group was 31-40 years with five (10.0%) subjects (Table 1).

**Table 1:** Demographic distribution of the study population. Data are presented as N (%) for categorical variables and mean ± standard deviation (SD) for continuous variables. Percentages were calculated using the total study population (N = 50) as the denominator. SD, standard deviation.

Variable	Category	N (%)
Sex	Male	30 (60.0%)
	Female	20 (40.0%)
Age (years)	0–20	7 (14.0%)
	21–30	11 (22.0%)
	31–40	5 (10.0%)
	41–50	12 (24.0%)
	51–60	7 (14.0%)
	>60	8 (16.0%)
	Mean ± SD	41.84 ± 17.41

Clival anatomical variants were identified in 20 (40.0%) subjects. Among the identified variants, Canalis Basilaris Medianus (CBM) was the most frequently observed variant, present in 12 (24.0%) subjects. Craniopharyngeal Canal (CPC) and Fossa Navicularis Magna (FNM) were each identified in five (10.0%) subjects. All variants demonstrated a characteristic midline anatomical distribution on CBCT imaging (Table 2).

**Table 2:** Prevalence of clival anatomical variants (N = 50). Data are presented as N (%). Percentages were calculated using the total study population (N = 50) as the denominator. Descriptive statistical analysis was performed to determine the prevalence of clival anatomical variants.

Variant	N (%)
Canalis Basilaris Medianus (CBM)	12 (24.0%)
Craniopharyngeal Canal (CPC)	5 (10.0%)
Fossa Navicularis Magna (FNM)	5 (10.0%)
Any clival variant	20 (40.0%)

Sex-wise analysis demonstrated that CBM was more frequently observed among male subjects (8, 26.7%) compared to female subjects (4, 20.0%). CPC showed a relatively higher prevalence among female subjects (3, 15.0%) than male subjects (2, 6.7%). FNM was observed only among male subjects in the present sample (5, 16.7%), whereas no cases were identified among female subjects. However, statistical analysis revealed no significant association between sex and the occurrence of CBM (p = 0.624), CPC (p = 0.345), or FNM (p = 0.051) (Table 3).

**Table 3:** Distribution of clival variants by sex. Data are presented as N (%). Percentages were calculated within each sex category (Male, N = 30; Female, N = 20). Chi-square (χ²) test was used to assess the association between sex and the occurrence of clival anatomical variants. CBM, Canalis Basilaris Medianus; CPC, Craniopharyngeal Canal; FNM, Fossa Navicularis Magna; χ², Chi-square value. A p-value <0.05 was considered statistically significant. Given the small number of positive cases, sex-specific findings should be interpreted with caution.

Variant	Male (N = 30)	Female (N = 20)	χ² value	p-value
CBM	8 (26.7%)	4 (20.0%)	0.24	0.624
CPC	2 (6.7%)	3 (15.0%)	0.89	0.345
FNM	5 (16.7%)	0 (0.0%)	3.81	0.051

Analysis of clival variants across different age groups demonstrated that CBM showed a relatively uniform distribution across age categories. CPC was most frequently observed among subjects aged >60 years (3, 37.5%), and a statistically significant association was observed between age group and CPC prevalence (χ² = 12.14, p = 0.033). FNM was identified across several age groups, including subjects older than 60 years; however, the small number of cases precludes meaningful conclusions regarding age predilection. The highest overall prevalence of clival variants was observed among subjects aged >60 years, in whom six (75.0%) subjects demonstrated at least one variant (Table 4).

**Table 4:** Age-wise distribution of clival variants. Data are presented as N (%). Percentages were calculated within each age group category. Chi-square (χ²) test was used to evaluate the association between age groups and prevalence of clival anatomical variants. CBM, Canalis Basilaris Medianus; CPC, Craniopharyngeal Canal; FNM, Fossa Navicularis Magna; χ², Chi-square value. A p-value <0.05 was considered statistically significant. *Statistically significant. Age-group findings should be interpreted cautiously because of the limited sample size within individual age categories.

Variant	0–20 years (N = 7)	21–30 years (N = 11)	31–40 years (N = 5)	41–50 years (N = 12)	51–60 years (N = 7)	>60 years (N = 8)	χ² value	p-value
CBM	2 (28.6%)	2 (18.2%)	1 (20.0%)	4 (33.3%)	2 (28.6%)	1 (12.5%)	2.94	0.709
CPC	0 (0.0%)	0 (0.0%)	1 (20.0%)	1 (8.3%)	0 (0.0%)	3 (37.5%)	12.14	0.033*
FNM	1 (14.3%)	1 (9.1%)	1 (20.0%)	0 (0.0%)	0 (0.0%)	2 (25.0%)	7.86	0.164
Any clival variant	2 (28.6%)	3 (27.3%)	2 (40.0%)	5 (41.7%)	2 (28.6%)	6 (75.0%)	6.51	0.260
No variant	5 (71.4%)	8 (72.7%)	3 (60.0%)	7 (58.3%)	5 (71.4%)	2 (25.0%)	6.51	0.260

Among the 20 subjects demonstrating clival anatomical variants, 18 (90.0%) subjects exhibited a single variant, whereas co-occurrence of two variants was observed in two (10.0%) subjects. CBM alone was the most commonly encountered isolated variant, accounting for 10 (50.0%) positive cases. CPC alone and FNM alone were each identified in four (20.0%) subjects. Simultaneous occurrence of CBM with CPC and CBM with FNM was observed in one subject each, whereas no subject demonstrated concurrent presence of all three variants (Table 5).

**Table 5:** Co-occurrence pattern of clival variants among variant-positive subjects (N = 20). Data are presented as N (%). Percentages were calculated using only variant-positive subjects (N = 20) as the denominator. Descriptive statistical analysis was performed to assess the co-occurrence pattern of clival anatomical variants. CBM, Canalis Basilaris Medianus; CPC, Craniopharyngeal Canal; FNM, Fossa Navicularis Magna.

Combination	N (%) among positive cases (N = 20)
CBM alone	10 (50.0%)
CPC alone	4 (20.0%)
FNM alone	4 (20.0%)
CBM + CPC	1 (5.0%)
CBM + FNM	1 (5.0%)
FNM + CPC	0 (0.0%)
All three variants	0 (0.0%)

## Discussion

The present study evaluated the prevalence and demographic distribution of three clival anatomical variants, namely Canalis Basilaris Medianus (CBM), Craniopharyngeal Canal (CPC), and Fossa Navicularis Magna (FNM), using large field-of-view CBCT scans obtained from a tertiary care dental institution in South India. Clival anatomical variants were identified in 20 (40.0%) subjects, indicating that these findings may be encountered during routine dental CBCT evaluation. However, the prevalence observed in the present study should be interpreted cautiously because of the relatively small sample size and single-center design. Nevertheless, the findings underscore the importance of systematic skull base assessment during CBCT interpretation. Akkoca Kaplan et al. reported a substantially higher prevalence of clival variants in a Turkish population using CBCT imaging, with CBM observed in 31.6%, FNM in 27.2%, and CPC in 8.1% of subjects [6]. In contrast, Bayrak et al. reported lower prevalences of FNM (5.6%), CBM (2.5%), and CPC (0.3%) using CT and CBCT imaging [7]. Variations in prevalence among studies may be attributed to differences in imaging modalities, study design, sampling methods, diagnostic criteria, and population characteristics.

CBM was the most frequently identified clival variant in the present study, observed in 12 (24.0%) subjects. CBM is considered the most common developmental osseous canal involving the clivus and is believed to originate either from persistence of embryonic vascular channels or remnants of the notochord [8,9]. On CBCT imaging, it typically appears as a well-corticated midline canal within the basiocciput and may occasionally mimic osteolytic lesions or traumatic defects involving the clivus [10]. In the present study, CBM demonstrated a relatively uniform age distribution. No statistically significant association was identified between CBM prevalence and age or sex. A slightly higher frequency was observed among male subjects (8, 26.7%); however, this finding should be interpreted cautiously because of the limited sample size. Similar observations were reported by Darwin et al. in an Indian CBCT-based study [9]. The developmental and clinical significance of CBM has also been emphasized in previous reports describing its association with recurrent meningitis and intracranial infection in pediatric patients [11,12].

CPC was identified in five (10.0%) subjects and demonstrated a statistically significant association with age group (p = 0.033), with the highest occurrence observed among subjects aged >60 years (3, 37.5%). CPC represents incomplete obliteration of the embryological tract of Rathke’s pouch and may establish communication between the nasopharynx and sellar region [13,14]. Previous studies have reported its association with cerebrospinal fluid leakage, recurrent infection, meningoceles, encephaloceles, and ectopic pituitary tissue [15-20]. CPC is therefore clinically significant during transsphenoidal and nasopharyngeal surgical procedures. In the present study, CPC demonstrated a slightly higher prevalence among female subjects (3, 15.0%) compared with male subjects (2, 6.7%), although this association was not statistically significant. While a significant age-group association was observed, the number of CPC-positive cases was small, and therefore, this finding requires confirmation in larger studies before definitive conclusions can be drawn.

FNM was identified in five (10.0%) subjects. All FNM cases in the present sample occurred among male subjects; however, no statistically significant association with sex was observed. FNM is generally described as a notch-like corticated defect along the inferior aspect of the clivus and is believed to arise from persistence of emissary venous channels or notochordal remnants [21,22]. Similar to other clival variants, FNM may mimic fractures or destructive lesions on imaging and may also serve as a potential pathway for the spread of infection from the nasopharynx to the intracranial compartment [23]. FNM was identified across several age groups, including subjects older than 60 years; however, the small number of cases precludes meaningful conclusions regarding age predilection. Co-occurrence of clival variants was observed in two (10.0%) positive subjects, with both cases involving CBM. This finding may support the possibility of shared developmental mechanisms involving persistence of embryological tracts and incomplete fusion of basioccipital ossification centers; however, further studies are required to clarify these relationships.

Strengths and limitations

The present study is among the few CBCT-based investigations evaluating clival anatomical variants in an Indian population. Use of large field-of-view CBCT scans enabled comprehensive visualization of the skull base and clival region, facilitating identification of incidental anatomical variants that may otherwise remain undetected during routine dental imaging. Accurate recognition of variants such as CBM, CPC, and FNM is clinically important to avoid misdiagnosis as pathological lesions or fractures and to assist in appropriate radiological interpretation and preoperative planning. Furthermore, all scans were independently evaluated by two experienced oral radiologists with repeated assessment at separate time intervals, thereby improving diagnostic reliability and minimizing observer variability. Furthermore, interobserver agreement between the two oral radiologists was excellent (Cohen's κ = 0.87), supporting the reliability and consistency of the radiographic assessments. The findings of the present study reinforce the importance of systematic skull base evaluation during routine CBCT interpretation.

The present study has certain limitations. The sample size of 50 subjects provides limited statistical power for subgroup analyses and may increase the likelihood of type II error, particularly when evaluating associations according to age and sex. In addition, the relatively small number of positive cases for certain variants may have limited the precision of subgroup analyses and associated statistical comparisons. Additionally, the use of consecutive sampling from a single institutional archive may not accurately represent the broader population. As the study was retrospective and imaging-based in nature, correlation with clinical symptoms, surgical findings, and patient outcomes could not be performed. Furthermore, the study was conducted at a single center in South India, thereby limiting the generalizability of the findings. Consequently, the prevalence estimates reported in the present study should be interpreted as institution-based observations rather than population-level estimates. Larger multicentric studies with broader geographic representation are required to establish more representative prevalence data and to better understand the clinical significance of clival anatomical variants.

## Conclusions

Clival anatomical variants such as Canalis Basilaris Medianus, Craniopharyngeal Canal, and Fossa Navicularis Magna were identified in a proportion of archived large field-of-view CBCT scans evaluated in the present study and represent important incidental findings that may be encountered during routine radiographic assessment. Adequate knowledge regarding the imaging appearance and anatomical characteristics of these variants is essential for dental practitioners and oral radiologists to avoid misdiagnosis as pathological lesions or fractures and to prevent unnecessary clinical or surgical intervention. The findings of the present study highlight the value of systematic evaluation of the skull base region during CBCT interpretation to facilitate accurate diagnosis, appropriate referral, and preoperative planning when indicated.

Given the relatively small sample size and single-center design, the prevalence estimates reported in this study should be interpreted with caution. Further multicentric studies with larger sample sizes are warranted to better characterize the prevalence and clinical significance of clival anatomical variants in diverse populations.
